# DAEiS-Net: Deep Aggregation Network with Edge Information Supplement for Tunnel Water Stain Segmentation

**DOI:** 10.3390/s24175452

**Published:** 2024-08-23

**Authors:** Yuliang Wang, Kai Huang, Kai Zheng, Shuliang Liu

**Affiliations:** 1 Beijing Metro Construction Administration Co., Ltd., Beijing 100068, China; 2 Beijing Key Laboratory of Fully Automatic Operation and Safety Monitoring for Urban Rail Transit, Beijing 100068, China; 3 College of Civil Engineering, Tongji University, Shanghai 200092, China; 4 Beijing MTR Corporation Ltd., Beijing 100068, China; 5 School of Advanced Manufacturing Engineering, Chongqing University of Posts and Telecommunication Technology, Chongqing 400065, China

**Keywords:** semantic segmentation, tunnel disease, water stain segmentation, attention mechanism, multiscale fusion

## Abstract

Tunnel disease detection and maintenance are critical tasks in urban engineering, and are essential for the safety and stability of urban transportation systems. Water stain detection presents unique challenges due to its variable morphology and scale, which leads to insufficient multiscale contextual information extraction and boundary information loss in complex environments. To address these challenges, this paper proposes a method called Deep Aggregation Network with Edge Information Supplement (DAEiS-Net) for detecting tunnel water stains. The proposed method employs a classic encoder–decoder architecture. Specifically, in the encoder part, a Deep Aggregation Module (DAM) is introduced to enhance feature representation capabilities. Additionally, a Multiscale Cross-Attention Module (MCAM) is proposed to suppress noise in the shallow features and enhance the texture information of the high-level features. Moreover, an Edge Information Supplement Module (EISM) is designed to mitigate semantic gaps across different stages of feature extraction, improving the extraction of water stain edge information. Furthermore, a Sub-Pixel Module (SPM) is proposed to fuse features at various scales, enhancing edge feature representation. Finally, we introduce the Tunnel Water Stain Dataset (TWS), specifically designed for tunnel water stain segmentation. Experimental results on the TWS dataset demonstrate that DAEiS-Net achieves state-of-the-art performance in tunnel water stain segmentation.

## 1. Introduction

Urban transportation systems have become the lifeline for billions in today’s bustling metropolises, driving the heartbeat of cities and fostering urban development. As urban areas continue to expand, the demand for reliable transportation infrastructure has reached unprecedented levels. Tunnels are critical components of these systems, providing commuters with fast and convenient transportation options [1,2]. The safety and stability of tunnels, as essential components of transportation and urban infrastructure, is directly linked to the sustainable development of the socioeconomic landscape. Due to complex geological conditions, diverse construction techniques, and natural and human factors in long-term operations, tunnel structures are prone to various diseases such as cracks, leaks, spalling, and deformation [3,4,5]. If these diseases are not detected and repaired promptly, they may pose serious safety risks and even lead to catastrophic accidents; therefore, disease detection and maintenance in tunnels have become critical and urgent tasks in the field of engineering.

Traditional structural disease detection methods often rely on on-site manual inspections, which are time-consuming and labor-intensive [6,7,8]. Various non-destructive methods, such as acoustic emission [9,10,11,12,13], visual imaging [14,15,16], and ultrasonic tomography [17], have been employed for tunnel detection to enhance efficiency. Additionally, Order–Frequency Holo-Hilbert spectral analysis has been used to demodulate fault information in time-varying vibration signals [18]. However, these methods require manual parameter adjustments, and their detection effectiveness is often suboptimal. To address these issues, researchers have increasingly focused on machine vision approaches. With advancements in information technology, automated detection techniques based on image processing and computer vision have become research hotspots. In particular, the rapid development of deep learning technologies has provided novel solutions for tunnel disease detection. Deep learning models, particularly Convolutional Neural Networks (CNNs) [19,20,21], have demonstrated outstanding performance in tasks such as image classification, object detection, and image segmentation [22,23,24]. These models can automatically learn and extract complex features from images, enabling accurate identification and segmentation of tunnel diseases.

As machine learning [25,26,27] and deep learning [28,29,30] technologies develop, deep learning-based tunnel disease detection tasks can be primarily divided into two categories, namely, object detection and semantic segmentation. In the domain of object detection, several noteworthy studies have been conducted; for instance, Cha et al. [31] proposed a vision-based method that utilizes CNNs to detect concrete cracks without computing disease features. The designed CNN network was trained on 40,000 images, achieving an accuracy of 98%. Zhou et al. [32] introduced a YOLOv4-based model that incorporates depthwise separable convolution, enhancing the recognition accuracy of disease targets under complex tunnel backgrounds and lighting conditions. Li et al. [33] proposed a multi-scale disease region proposal network capable of generating disease region proposals on multilayer feature maps, with each layer focusing on diseases within a certain scale range. This multiscale detector ultimately improves the accuracy of disease detection. Li et al. [34] leveraged deep learning and computer vision to identify underwater structural damage in hydraulic tunnels. A high-performance detector was constructed based on YOLOv5 and an adaptive spatial feature fusion module. Through experiments, they determined the sparsity ratio and pruning rate, balancing accuracy and efficiency. Liu et al. [35] introduced a belief capsule network with a multi-stream capsule routing strategy and a consistency-aware fusion strategy to generate high-quality pseudolabels to solve the problem of deep supervised significant object detection being heavily dependent on labeling.

In the field of semantic segmentation, Chun et al. [36] proposed a supervised dynamic crack detection method. By considering the pixel values of target pixels and the geometric features of cracks when they are linearly connected, they introduced a segmentation method based on pixel values and geometric shapes. This method is applicable for tunnel crack segmentation under adverse lighting conditions such as the presence of shadows and dirt. Zhou et al. [37] addressed the characteristics of cracks and linear seams by proposing a Mixed Attention (MA) module based on effective embedding channels and positional information. Unlike common spatial attention modules that aggregate information across the entire space, the MA module aggregates features directly along the spatial dimensions of height and width. Liao et al. [38] introduced a novel mobile tunnel inspection system comprising a unique mobile imaging module and an automatic crack detection module. A novel lightweight CNN was utilized for tunnel crack detection, incorporating effective spatial constraint strategies to maintain the continuity of cracks.

Tunnel water stain disease segmentation, a specific type of tunnel disease detection, is particularly challenging due to the complexity and variability of the tunnel environment. Insufficient lighting inside tunnels often leads to images with low contrast and with significant noise. Additionally, water stains vary widely in morphology and can appear on different materials and surfaces, resulting in diverse visual features that are challenging to capture. Consequently, accurate segmentation of tunnel water stain diseases faces two main challenges: (1) significant variation in the morphology and size of different types of disease, which leads to insufficient extraction of multiscale contextual information; and (2) complex environmental and lighting conditions, which cause the loss of boundary detail information.

To address these challenges, this paper proposes a novel approach leveraging deep aggregation features supplemented with edge information. The proposed method, named Deep Aggregation Network with Edge Information Supplement (DAEiS-Net), adopts a classic encoder–decoder architecture for tunnel water leakage detection. Specifically, we introduce the Deep Aggregation Module (DAM) in the encoder section to enhance the network’s feature representation capabilities. To extract multiscale contextual information of water leakage and mitigate the effects of variable morphology and size, a Multiscale Cross-Attention Module (MCAM) is proposed. This module integrates spatial and semantic information, reducing noise in shallow features and enhancing texture information extraction. Additionally, to address inadequacies in extracting water leakage edge information, the Edge Information Supplement Module (EISM) is designed to bridge the semantic gaps at different stages, effectively integrating precise detailed information with rich semantic content. Furthermore, in the Sub-Pixel Decoder (SPD) is utilized as a Sub-Pixel Module (SPM) to combine features at various scales, improving the representation of edge features. Finally, we introduce the Tunnel Water Stain Dataset (TWS), a dataset specifically designed for tunnel water stain segmentation, covering various tunnel water leakage scenarios. Experimental results on the TWS dataset demonstrate that DAEiS-Net achieves state-of-the-art performance. The main contributions of this paper are summarized as follows:

1. A novel tunnel water leakage segmentation network model called DAEiS-Net is proposed, focusing primarily on aggregating multiscale contextual information and extracting edge information. By enhancing multiscale fusion, the segmentation results are significantly improved.
2. The proposed DAM incorporates MCAM to adaptively extract the multiscale contextual information of water leakage. The EISM is designed to integrate features from various stages, effectively bridging semantic gaps and enhancing edge information extraction. The SPD module is used in the decoding process to enhance edge feature representation.
3. The TWS dataset is developed to encompass a wide range of tunnel water leakage scenarios. This dataset provides a solid foundation for training and evaluating models.

The remainder of this paper is organized as follows: in Section 2, we review related work on tunnel water leakage segmentation; Section 3 provides a detailed description of our proposed method; in Section 4, we evaluate the effectiveness of our method; finally, Section 5 concludes the paper.

## 2. Related Work

### 2.1. Semantic Segmentation

Semantic segmentation algorithms directly separate relevant pixels from the background and visually display the predicted diseases in the image. Long et al. [39] proposed the first Fully Convolutional Network (FCN), which converts all fully connected layers in CNNs into convolutional layers and achieves semantic segmentation through pixel-wise classification. To address the slow speed of multistage methods and the low accuracy of single-stage methods, Ronneberger et al. [40] introduced the classic U-Net, a U-shaped encoder–decoder network. This method utilizes an encoder for downsampling to extract high-level image features and a decoder for upsampling to restore image details and spatial dimensions, resulting in excellent outcomes. To address the issue of network spatial inconsistency, Zhao et al. [41] proposed PSPNet, which utilizes a Spatial Pyramid Pooling (SPP) module to fuse multiscale contextual information, thereby reducing the probability of mis-segmentation inherent in FCNs. In order to capture richer multiscale contextual semantic information, Chen et al. [42] introduced DeepLabv1, which employs atrous convolution to overcome the issues of reduced image resolution and irrecoverable position information caused by repeated pooling and down-sampling. Chen et al. [43] later proposed SoCo, which utilizes the concept of self-supervised contrastive learning. Self-supervised constrastive learning extracts features by learning the similarity relationships between different parts of an image, achieving more accurate and robust natural semantic segmentation in images. However, due to multiple downsampling operations during feature extraction, existing algorithms lose a great deal of detail in the features, resulting in absence of effective local contextual information. Additionally, object edges are often segmented inaccurately due to incomplete feature fusion and insufficient global contextual information.

### 2.2. Segmentation Models Applied to Tunnel Disease Detection

Yang et al. [44] proposed a method based on U-Net for welding disease segmentation, utilizing an attention-guided segmentation network. To mitigate the loss of contextual information caused by multiple convolution and pooling operations, a multiscale feature fusion module was integrated into the U-Net to capture more comprehensive information. In response to the challenges of complex environmental interference and multiscale target recognition in tunnel disease identification, Zhou et al. [45] introduced a novel segmentation algorithm called Multiscale Attention and Context Information Enhancement. This algorithm was designed to create a context-enhanced feature encoder to address the limitations of CNNs. It aims to fully extract global contextual information and reduce false detections and omissions caused by complex environmental interference. To address the issue of small crack segmentation, Chu et al. [46] proposed a multiscale feature fusion network with an attention mechanism, which they named Tiny-Crack-Net (TCN). This network utilizes an enhanced residual network to capture local features of small cracks, and incorporates dual attention modules into the architecture to effectively distinguish tiny cracks from the background. Qin et al. [47] introduced a disease segmentation model based on Vision Transformer (ViT), which significantly differs from traditional CNNs. An adapter and decoding head were proposed to enhance the training effectiveness of the transformer encoder, enabling it to adapt to small-scale datasets.

## 3. Methodology

**Figure 1:**
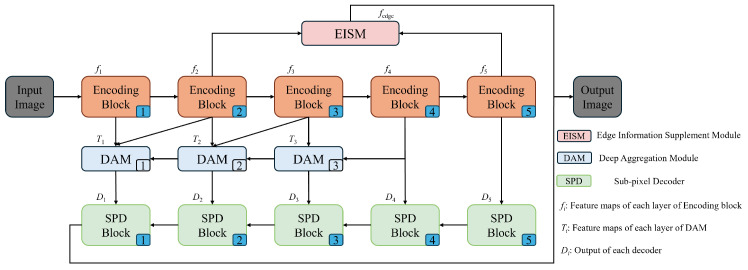
The schematic diagram of the DAEiS-Net structure, which is composed of encoding blocks, SPD blocks, EISM, and DAM.

The overall structure of the proposed method is illustrated in Figure 1. It adopts the encoder–decoder framework of U-Net, with ResNet-34 as the backbone network. In the encoder part, we introduce the DAM to enhance the representation capability of the network. The DAM incorporates an MCAM to fuse the semantic and spatial information, improving the model’s ability to extract multiscale information of tunnel water leakage and alleviating the impact of variable morphology and scale. Moreover, an EISM is employed to bridge the semantic gaps at different stages. The EISM effectively integrates precise detail information with rich semantic information to address the insufficiency in extracting the boundary information of tunnel water leakage. In the SPD decoder, an SPM is utilized to merge features at different scales, enhancing the representation of edge features. The specific convolution settings for each module in our proposed network are shown in Table 1.

### 3.1. Deep Aggregation Module

Different types of water leakage diseases vary significantly in morphology and scale. Therefore, enhancing the encoder’s ability to extract multiscale features is crucial to solving the problem of losing information on tunnel water leakage detection. The MCAM suppresses the noise in the shallow features and enhances the texture information of the high-level features by fusing the spatial and semantic information of different stages.

**Figure 2:**
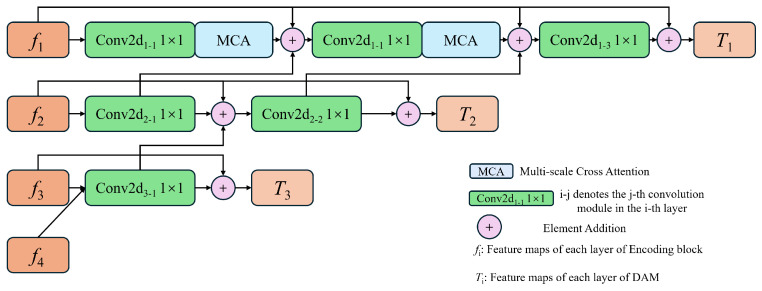
The schematic diagram of the DAM structure. The fused features are obtained through the upsampling operation. The final output is obtained through the MCAM.

The structure of the DAM is illustrated in Figure 2. In the deep feature layers, the fourth-layer feature $f_{4}$ is first upsampled. The upsampling operation consists of two standard convolutions (with a kernel size of 3) and bilinear interpolation with a scale factor of 2. Then, $f_{4}$ is convolved with $f_{3}$ to obtain the fused features. This convolution operation includes a 1×1 convolution (with both kernel size and stride of 1), batch normalization (BN), and ReLU activation. Finally, $f_{3}$ is concatenated with the fused features to form a residual connection, preventing gradient vanishing and yielding the final output $T_{3}$. This process is repeated for each layer in the encoder to obtain deep features $T_{2}$. The computation process is described by the following formulas:

(1) $$T_{3}=t_{3}+f_{3}=C_{3-1}\left(\left[f_{3},↑f_{4}\right]\right)+f_{3}$$

where + denotes matrix addition, $\left[\cdot \right]$ indicates concatenation, ↑ represents upsampling, and $C_{i-j}$ denotes the *j*-th convolution module in the *i*-th layer. Here, $t_{i}$ represents the fused features of $f_{i}$ and the upsampled $f_{i+1}$ of the *i*-th layer. The DAM fuses features from adjacent layers, where the deep feature is used to extract rich semantic information and the intermediate layer features contain edge texture information and semantic information. The network can extract the multiscale information of tunnel water leakage based on the fused features in an adaptive manner.

Specifically, as shown in Figure 2, we integrate the MCAM at the feature fusion stage of the first layer. We fuse $f_{i+1}$ with the upsampled $f_{2}$, then the fused features are enhanced using the MCAM to obtain the fused feature $t_{1-1}$. Subsequently, a second convolution operation and the MCAM are applied to further fuse $f_{i+1}$, $t_{1-1}$, and $t_{2-1}$. Based on this, the final output $T_{1}$ is obtained. In this way, fused features with rich texture information and semantic information are achieved through continuous feature fusion operations.

Algorithmically, this can be defined as

(2) $$t_{1-1}=A\left(C_{1-1}\left(\left[f_{1},↑f_{2}\right]\right)\right),$$

(3) $$t_{1-2}=A\left(C_{1-2}\left(\left[f_{1},t_{1-1},↑t_{2-1}\right]\right)\right),$$

(4) $$T_{1}=t_{1-3}+f_{1}=C_{1-3}\left(\left[f_{1},t_{1-2},↑t_{2-2}\right]\right)+f_{1},$$

where $t_{1-1}$ represents the feature map obtained by fusing $f_{1}$ and $f_{2}$, $T_{1}$ denotes the output of the first layer, and A denotes the entire process of the MCAM. We propose the MCAM to suppress the noise in the shallow features and enhance the feature representation capability of the model. Additionally, the MCAM improves the connectivity structure of the network, allowing for more efficient utilization of the neurons in the neural network for enhanced computational efficiency and overall network performance.

**Figure 3:**
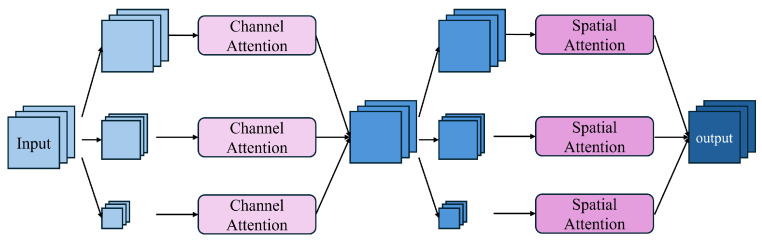
The schematic diagram of the MCAM structure. The MCAM comprises multiple convolutional blocks of different scales along with channel attention and spatial attention mechanisms.

As illustrated in Figure 3, the MCAM comprises multiple convolutional blocks of different scales along with spatial attention and channel attention mechanisms. The MCAM performs attention operations on the features at various scales in order to adaptively extract the relevant information while suppressing the noise. Specifically, the input features undergo convolution operations followed by 2× and 4× downsampling. These features are then fed into the channel attention mechanism at three different scales. Subsequently, a convolution operation is applied to integrate the multiscale features, obtaining the intermediate features. These intermediate features are downsampled by 2× and 4× and then processed by the spatial attention mechanism. The final output features are obtained through a convolution operation.

**Figure 4:**
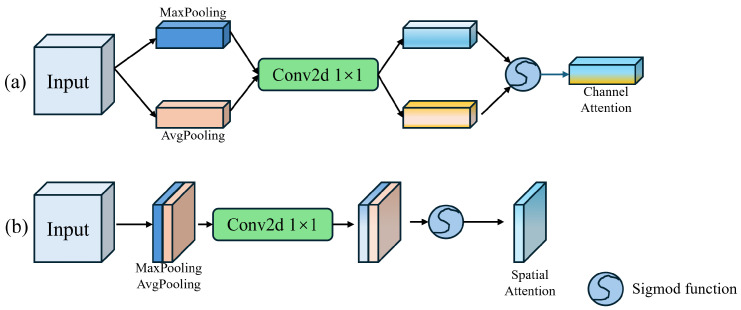
The structure of channel attention (**a**) and spatial attention (**b**). The attention operators are obtained through max pooling and the global average pooling.

The process of channel attention is depicted in Figure 4a. Initially, the input features undergo global max pooling and global average pooling operations to obtain two different feature descriptor operators. These operators are then processed by a 1×1 convolution to perform dimensionality reduction and expansion, enhancing the feature representation in the attention map. Finally, the channel weights are normalized using the sigmoid function. The output feature map is produced by applying the channel weights to the input feature map through a residual connection.

The process of spatial attention is illustrated in Figure 4b. Initially, the feature map processed by channel attention undergoes max pooling and average pooling operations along the channel dimension. The two resulting feature tensors are concatenated and passed through a 1×1 convolution to adjust the number of channels. The spatial attention map is then normalized using the sigmoid function. Finally, the spatial attention operator is combined with the input feature map through a residual connection to produce the final output of the MCAM.

The MCAM enhances the network’s ability to perceive objects and resist interference by effectively integrating and filtering multiscale features. The MCAM can adaptively weight each feature of different scales using attention mechanisms, which improves the representational capability of the model. Furthermore, the MCAM captures detailed features of objects at various scales, enhancing the network’s detection accuracy and localization precision.

### 3.2. Edge Information Supplement Module

The interiors of tunnels often suffer from insufficient lighting, resulting in images with low contrast but significant noise. Additionally, diseases exhibit diverse shapes and appear on different materials and surfaces, making their visual characteristics varied and difficult to capture. Inadequate extraction of water leakage edge information leads to suboptimal segmentation results. To address this problem, we employ an EISM to enhance the extraction of edge information by directly supervising the exploration of edge semantic information.

**Figure 5:**
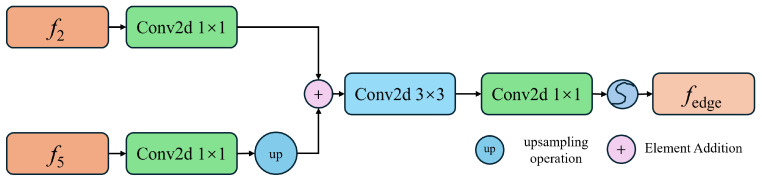
The structure diagram of the edge information module. The fused features are concatenated along the channel dimension.

In deep learning networks, shallow features contain extensive regional texture information, including edge interference; in contrast, deep features contain rich semantic information, which can be used to locate relevant edges within the shallow features. The designed EISM exploits the edge information from $f_{1}$ and $f_{4}$. As shown in Figure 5, the structure of the edge information module is as follows: first, features from both layers are input into 1×1 convolutions to balance the proportion of high-level semantic information. The features of $f_{4}$ are upsampled to match the spatial resolution of $f_{2}$. Then, the features from both layers are concatenated along the channel dimension and further integrated with a 3×3 convolution to enhance the semantic information of the feature map, which strengthens the edge information extraction capability. Finally, a 1×1 convolution followed by a sigmoid activation function produces the edge information segmentation result.

### 3.3. Sub-Pixel Decoder

In traditional encoder–decoder architectures, most methods rely on upsampling and skip connections to restore the resolution of the feature maps in the decoder. However, upsampling often leads to inevitable information loss. To address this problem, we propose an SPD to enhance feature representation and minimize information loss during feature fusion, which improves the accuracy of water stain segmentation.

**Figure 6:**
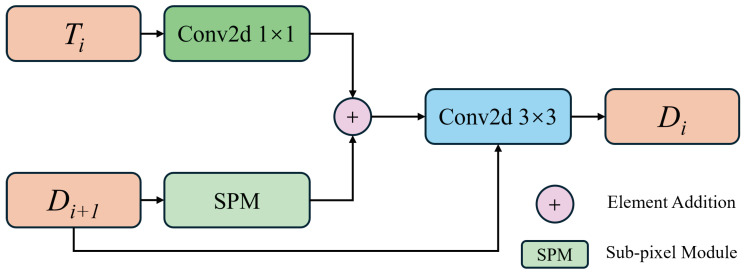
The SPD structure diagram; $D_{i}$ is updated by the SPM and convolution operation.

The SPD is primarily composed of four sub-pixel convolution modules, as shown in Figure 6. Initially, $D_{4}$ is upsampled via sub-pixel operations to match the size of the third layer network. The $T_{3}$ features undergo a convolution for dimensionality reduction, which reduces the number of parameters and enhances the efficiency of the network. Subsequently, both features are fused after passing through separate 3×3 convolutions. The fused features are then added pixel-wise to the output from the sub-pixel upsampling. A final 3×3 convolution yields the decoder’s output $D_{3}$. This process is repeated similarly to obtain the output $D_{i}$ for each subsequent layer in the decoder.

In algorithmic form, this is described as

(5) $$E_{i}^{,}=\left(C_{3}\left(C_{1}\left(E_{i}\right)\right)\right),$$

(6) $$D_{i}=C_{3}\left(C_{3}\left(SPM\left(D_{i+1}\right)\right)\times E_{i}^{,}\right)+SPM\left(D_{i+1}\right)),$$

where $D_{i}$ represents the output of the decoder, $C_{1}$ denotes the convolution operation with a kernel size of 1 × 1, $E_{i}^{,}$ indicates the intermediate output, and SPM(·) represents the sub-pixel upsampling operation.

In conventional upsampling operations, bilinear interpolation is commonly used. This method calculates the target pixel value by taking the weighted average of the four nearest pixels in a 2×2 region around the corresponding source image position. However, bilinear interpolation has the drawback of causing blurring and distortion, especially in regions with high-frequency textures. This inadequacy can lead to insufficient extraction of tunnel water leakage boundary information. The reason for this is that bilinear interpolation only considers the local distribution of four neighboring pixels around the target pixel, neglecting the global information. As a result, when high-frequency textures surround the target pixel, bilinear interpolation can produce overly smooth results, leading to image distortion. In contrast, transposed convolution (deconvolution) upsampling often involves padding zeros in empty spaces when enlarging a small image, which introduces invalid information to the network.

**Figure 7:**
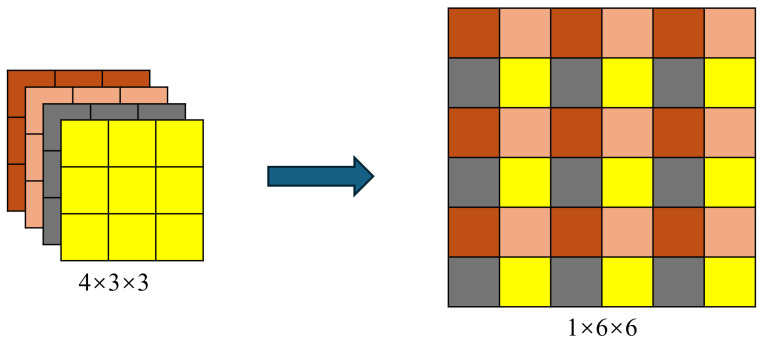
The process of subpixel upsampling. The channels are combined according to a specific pattern, resulting in a feature map that is twice the original size with one-fourth the number of channels.

In contrast to bilinear interpolation, sub-pixel upsampling performs convolution operations on the feature map and splits it into several groups along the channel dimension. The feature maps within each group are then rearranged to form a higher-resolution feature map. This method increases resolution while preserving more details, reducing information loss, and minimizing blurriness. In addition, it avoids the issues of high-frequency component damage and invalid padding, thereby enhancing the performance of semantic segmentation. The detailed process is as follows: for every four channels in the feature map, the channels are combined according to a specific pattern, resulting in a feature map that is twice the original size with one-fourth the number of channels, thereby achieving 2× upsampling. This process is illustrated in Figure 7.

### 3.4. Loss Function

In pixel-level segmentation tasks, the cross-entropy loss is commonly used to measure the discrepancy between the predictions of the model and the ground truth labels. For binary classification tasks, such as determining whether each pixel belongs to tunnel water leakage or not, the binary cross-entropy loss is utilized to evaluate the model’s accuracy. The calculation process is as follows:

(7) $$L_{bce}\left(P,G\right)=-\sum_{i=1}^{H}\sum_{j=1}^{W}\left(P_{ij}log\left(G_{ij}\right)+\left(1-P_{ij}\right)log\left(G_{ij}\right)\right)$$

where $G_{ij}$ and $P_{ij}$ represent the ground truth label and the predicted value at position (i,j), respectively.

Unlike the cross-entropy loss function, the Intersection over Union (IoU) loss is primarily used to measure the overlap between the predicted segmentation map and the ground truth labels, thereby accelerating the network’s optimization. The calculation process is as follows:

(8) $$L_{IoU}\left(P,G\right)=1-\frac{\sum_{i=1}^{H}\sum_{j=1}^{W}\left(G_{ij},P_{ij}\right)}{\sum_{i=1}^{H}\sum_{j=1}^{W}\left(G_{ij}+P_{ij}-G_{ij}\times P_{ij}\right)}.$$

The Structural Similarity (SSIM) loss is used to compare the Structural Similarity Index of two images. SSIM is a method for measuring the similarity between two images based on human visual perception, considering luminance, contrast, and structure. By calculating the SSIM index between two images, it is possible to assess their degree of similarity. The calculation process is as follows:

(9) $$\mathrm{SSIM}\left(x,y\right)=\frac{\left(2\mu_{x}\mu_{y}+M_{1}\right)\left(2\sigma_{xy}+M_{2}\right)}{\left(\mu_{x}^{2}+\mu_{y}^{2}+M_{1}\right)\left(\sigma_{x}^{2}+\sigma_{y}^{2}+M_{2}\right)}$$

where *x* and *y* represent the predicted result and the ground truth label, $\mu_{x}$ is the mean value of *x*, $\mu_{y}$ is the mean value of *y*, $\sigma_{x}^{2}$ is the variance of *x*, $\sigma_{y}^{2}$ is the variance of *y*, $\sigma_{xy}$ is the covariance between *x* and *y*, and $C_{1}$ and $C_{2}$ are constants used to stabilize the division, and are typically small values.

The overall loss function is calculated as follows:

(10) $$L\left(P,G\right)=L_{bce}\left(P,G\right)+L_{IoU}\left(P,G\right)+\mathrm{SSIM}\left(x,y\right).$$

## 4. Experiments

In this section, we present the experimental setup and discuss the comparative results of the proposed method against the current state-of-the-art algorithms.

### 4.1. Datasets

**Figure 8:**
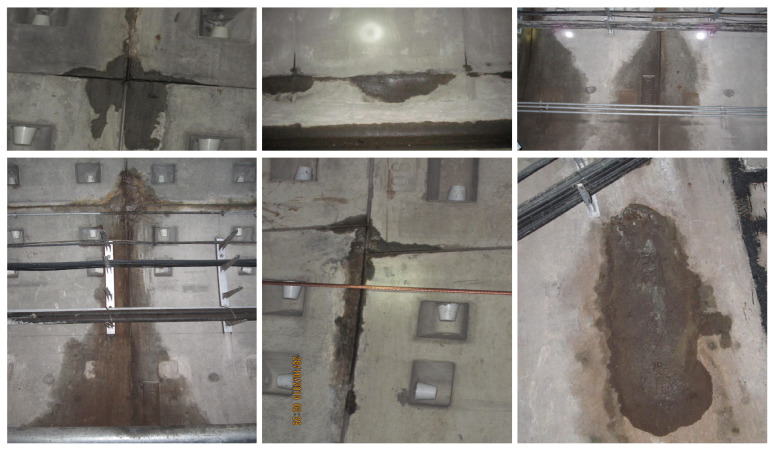
Different types of tunnel water stain samples in our dataset.

TWS Dataset: In this study, we created the TWS dataset, which was designed specifically for the task of tunnel water stain segmentation. This dataset aims to capture various instances of water stains within tunnels, providing a diverse set of training, validation, and testing samples to evaluate the effectiveness of our proposed method. The dataset details are shown in Table 2 and some samples are shown in Figure 8.

### 4.2. Evaluation Metrics

The segmentation of water stain within tunnels is fundamentally a pixel-level segmentation task; therefore, this study utilizes standard pixel-level evaluation metrics to assess the proposed method. These evaluation metrics include Accuracy (Acc), IoU, and F1-score [48,49,50,51]. Acc refers to the model’s pixel classification accuracy across all categories. The IoU measures the overlap between the predicted values of tunnel water stain and the ground truth labels, serving as a critical metric for evaluating pixel-level tasks. The F1-score is an important metric that balances the precision–recall relationship. The formulas for these metrics are as follows:

(11) $$Acc=\frac{TP+TN}{TP+FN+FP+TN},$$

(12) $$IoU=\frac{P_{p}\cap P_{g}}{P_{p}\cup P_{g}},$$

(13) $$F1-\mathrm{score}=\frac{2\times P\times R}{P+R},$$

where True Positive (TP), True Negative (TN), False Positive (FP), and False Negative (FN) represent the counts of true positive, true negative, false positive, and false negative pixels, respectively, while $P_{p}$ and $P_{g}$ denote the respective pixel values of the prediction and ground truth.

### 4.3. Experimental Setup

The proposed method was implemented in Python3.8 using the PyTorch framework. The experiments were conducted on a computer running the Ubuntu operating system with the following configuration: an Intel i7-7800X CPU (Santa Clara, CA, USA) and two NVIDIA GeForce RTX 2080Ti GPUs (Santa Clara, CA, USA) with 11 GB of memory each. During training, the Adam optimizer was employed with an initial learning rate of $10^{-4}$, a momentum of 0.9, and a weight decay of $10^{-5}$. The batch size was set to 32. If the IoU did not improve for five consecutive epochs, the learning rate was reduced by a factor of 0.2 until it reached zero, at which point the training process was terminated. Due to GPU memory limitations, all images used in the experiments were resized to a size of 512×512 pixels. To enhance the randomness of the input images, data augmentation strategies such as random cropping and horizontal and vertical flipping were applied during preprocessing. All experiments were conducted on the TWS dataset.

### 4.4. Experimental Results and Discussion

To evaluate the performance of the proposed method, we compared it against several state-of-the-art methods. These methods included classic semantic segmentation networks such as U-Net [40], PANet [52], CCNet [53], DeepLabV3+ [54], SCDeepLab [55], ViT-Seg [47], TCDNet [37], and MTIS [38]. All experimental results were generated based on open-source model implementations or models provided by the authors. It is important to note that the implementation settings for our method were consistent with the experimental details of all reproduced networks, including data augmentation strategies.

#### 4.4.1. Training Process

**Figure 9:**
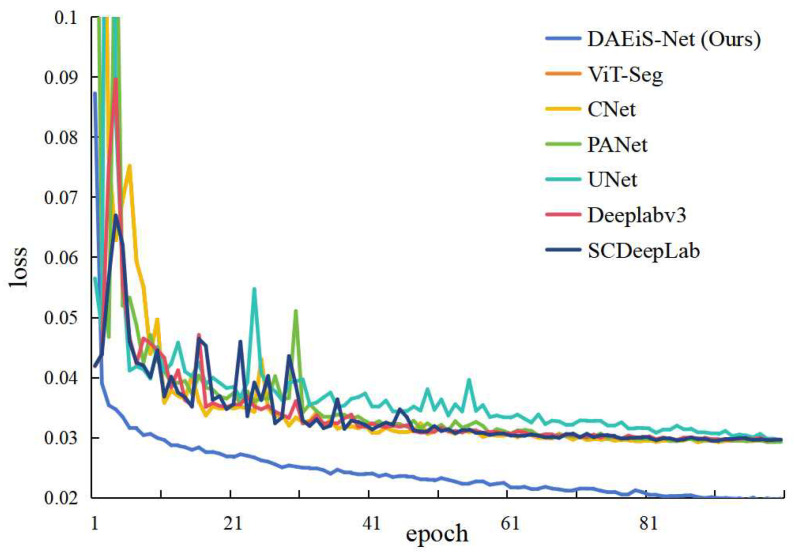
The loss curves during the training process; the horizontal axis represents the epoch, while the vertical axis represents the loss value.

We focused on the variation of the loss function during the training process. By analyzing the loss curves, the convergence speed and stability of each model can be intuitively understood. As shown in Figure 9, the loss curves of each model during training reveal significant insights. DAEiS-Net demonstrates rapid convergence speed from the initial stages of training. Compared to other methods, its loss value decreases rapidly and reaches a stable state within a relatively small number of iterations. This indicates that DAEiS-Net effectively learns useful features from the training data, thereby accelerating the optimization process. Moreover, DAEiS-Net maintains the lowest loss value throughout the training process, further demonstrating the superiority of the model. A low loss value indicates a smaller error between the model’s predictions and the ground truth, suggesting that DAEiS-Net achieves high accuracy and robustness in handling the tunnel water stain segmentation task.

In contrast, the loss curves of the other six methods demonstrate slower convergence speeds, and display significant fluctuations even in the later stages of training. This suggests that these methods face greater optimization challenges and instability when applied to tunnel water stain segmentation. By comparing the loss curves during the training process, it is evident that DAEiS-Net significantly outperforms the other methods in terms of both convergence speed and loss value. These observations underscore the effectiveness of DAEiS-Net in quickly learning and optimizing features essential for accurate and reliable tunnel water stain segmentation, emphasizing its potential for practical applications in demanding environments.

#### 4.4.2. Quantitative Comparison

In our experiments, we utilized common pixel-level metrics such as Acc, IoU, and F1-score to evaluate the proposed method. mAcc, mIoU and mF1 represent the mean values of Acc, IoU and F1-score respectively. The experimental results are presented in Table 3. Each row of the table represents the evaluation results of a different method, while each column corresponds to a specific evaluation metric.

The proposed model has a parameter count of 15.3 M, which is the lowest among all the listed models. In comparison, other models have parameter counts ranging from 25.8 M to 113.71 M, with the ViT-Seg model having the highest count at 113.71 M. A lower parameter count implies reduced computational resource requirements and faster inference speed, making the model suitable for deployment in resource-constrained environments. For the mean accuracy (mAcc) metric, the proposed model achieves an mAcc of 0.7923, which is second only to ViT-Seg’s mAcc of 0.8021. Despite having a lower parameter count, the proposed model achieves near-maximum accuracy, showcasing its strength in terms of precision. The proposed model also achieves a mean Intersection over Union (mIoU) of 0.7874, surpassing most other models. A higher mIoU indicates that the model excels in segmentation tasks by accurately predicting water stain regions. Additionally, the proposed model achieves an mF1-score of 0.77, matching DeepLabV3+, the highest among all models. This indicates that the proposed model not only effectively identifies water stain regions but also minimizes false positives.

The GAM in our model incorporates the MCAM to enhance feature extraction capabilities by integrating spatial and semantic information, reducing noise in shallow features, and enhancing texture extraction. This enhancement improves the model’s capability to detect water stains of different sizes. Furthermore, the proposed EISM bridges the semantic gap between different stages, effectively combining precise detailed information with rich semantic information to address deficiencies in extracting tunnel water stain boundary information. Despite having the fewest parameters, our model achieves near-optimal performance in mAcc and mIoU and attains the highest score in mF1, demonstrating its superiority in segmentation tasks. When compared to models with similar or even larger parameter counts, such as U-Net, PANet, and CCNet, the proposed model performs significantly better, highlighting its efficiency and effectiveness.

#### 4.4.3. Qualitative Comparison

**Figure 10:**
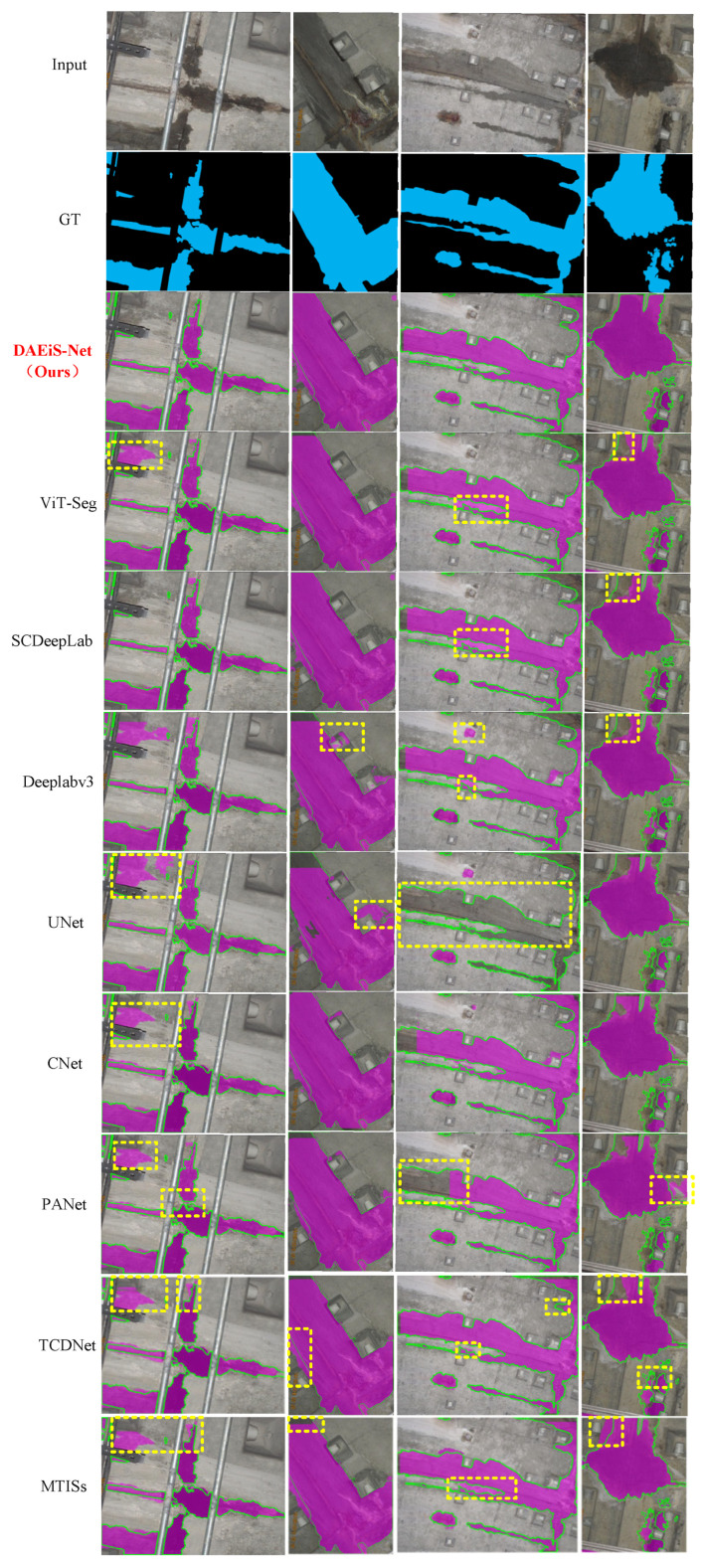
Qualitative analysis of the results. The first row shows the original images, the second row shows their corresponding ground truth values, and rows (3)–(9) show the segmentation results of our proposed model and the other classical models. The purple part is the segmentation area, and the yellow dotted line box represents the key contrast part of different methods.

To further demonstrate the effectiveness of our method, we conducted a qualitative analysis of the results generated by classical semantic segmentation networks. Figure 10 shows examples of tunnel water stain extraction from our dataset. We selected representative samples, including tunnel water stains in complex environments and those with varying scales. The first and second rows of Figure 10 show the original images and their corresponding ground truth values, respectively. Rows (3)–(9) display the segmentation results from our proposed model and other classical models. Compared to the other methods, our proposed model demonstrates superior performance in extracting tunnel water stains from various environments, areas, and shapes.

As shown in Figure 10, when traditional segmentation models such as DeepLabV3+, U-Net, CCNet, and PANet are applied to the task of tunnel water stain segmentation, their performance is inadequate; specifically, DeepLabV3+ and U-Net exhibit significant segmentation errors over large areas. In contrast, Vit-Seg and SCDeepLab, which are specifically designed for tunnel defect segmentation, perform better at this task. They tend to overlook more detailed boundary and structural information when handling tunnel water stain segmentation, which could lead to a decrease in segmentation accuracy in practical applications. However, these models often overlook detailed boundary and structural information. In comparison, the proposed model introduces an EISM, which bridges the semantic gap between different stages and enhances the network’s sensitivity to edge information, resulting in optimal extraction performance. Moreover, the GAM integrated with the MCAM strengthens the network’s feature extraction capability by combining spatial and semantic information. This integration reduces noise in shallow features and improves texture information extraction. Additionally, the proposed SPD enhances the network’s feature integration capability within the decoder.

In summary, the superior overall performance of our model can be attributed to the application of the GAM and MCAM. These modules enhance the feature extraction capability by integrating spatial and semantic information, effectively reducing noise in shallow features while reinforcing texture information extraction. This comprehensive approach ensures better handling of edge information and multiscale feature extraction, making our model highly effective for tunnel water stain segmentation tasks.

#### 4.4.4. Ablation Study

To further validate the impact of various modules in our proposed DAEiS-Net, we conducted an ablation study on the TWS dataset. It is important to highlight that compared to U-Net, our DAEiS-Net incorporates DAM, EISM, and SPD modules; the DAM includes the MCAM to enhance feature extraction in the encoder, the EISM is designed to improve the extraction of edges of tunnel water stains, and the SPD aims to enhance feature integration in the decoder. In order to systematically evaluate the effectiveness of each proposed module, we conducted several experiments. Initially, the baseline does not include the segmentation model based on the above functional modules.

As shown in Table 4, the baseline model has a parameter count of 13.2 M, achieving an mAcc of 0.7238, mIoU of 0.7001, and mF1 of 0.71. This baseline serves as the reference point for all improved models. Upon introducing the DAM module, the parameter count increases to 14.8 M, resulting in an mAcc improvement to 0.7652 and mIoU improvement to 0.7537, while mF1 remains at 0.71. This indicates that the DAM module significantly enhances the feature extraction capability of the encoder, thereby improving segmentation performance. With the inclusion of both the EISM and SPD modules in the baseline model the parameter count increases to 15.3 M, leading to an mAcc of 0.7856, mIoU of 0.7622, and mF1 of 0.75. Compared to solely introducing the DAM module, the inclusion of the EISM module notably enhances mAcc and mIoU, demonstrating its crucial role in improving tunnel water stain edge extraction. Introducing the DAM and SPD modules results in a parameter count of 15.3 M, resulting in an mAcc of 0.7731, mIoU of 0.7521, and mF1 of 0.74. Adding the SPD module alongside the DAM module enhances the feature integration capability of the decoder, significantly boosting mIoU and mF1 compared to solely introducing the DAM module. By sequentially introducing the DAM, EISM, and SPD modules on top of the baseline model, the parameter count remains at 15.3 M, with mAcc improving to 0.7923, mIoU to 0.7874, and mF1 to 0.77. Across all three metrics, the introduction of all modules results in performance improvements of 9.46%, 12.46%, and 8.45%, respectively.

The comprehensive integration of the DAM, EISM, and SPD modules in our proposed model leads to significant improvements across all performance metrics, with particularly noticeable enhancements in mIoU and mF1. These results indicate that the DAM module enhances feature extraction capability, the EISM module improves edge extraction performance, and the SPD module enhances feature integration capability. The combination of these three modules effectively addresses the challenges of multiscale boundary information loss and global information extraction in tunnel water stain segmentation tasks, outperforming both the baseline model and other individual or partial module combinations.

#### 4.4.5. Feature Visualization

**Figure 11:**
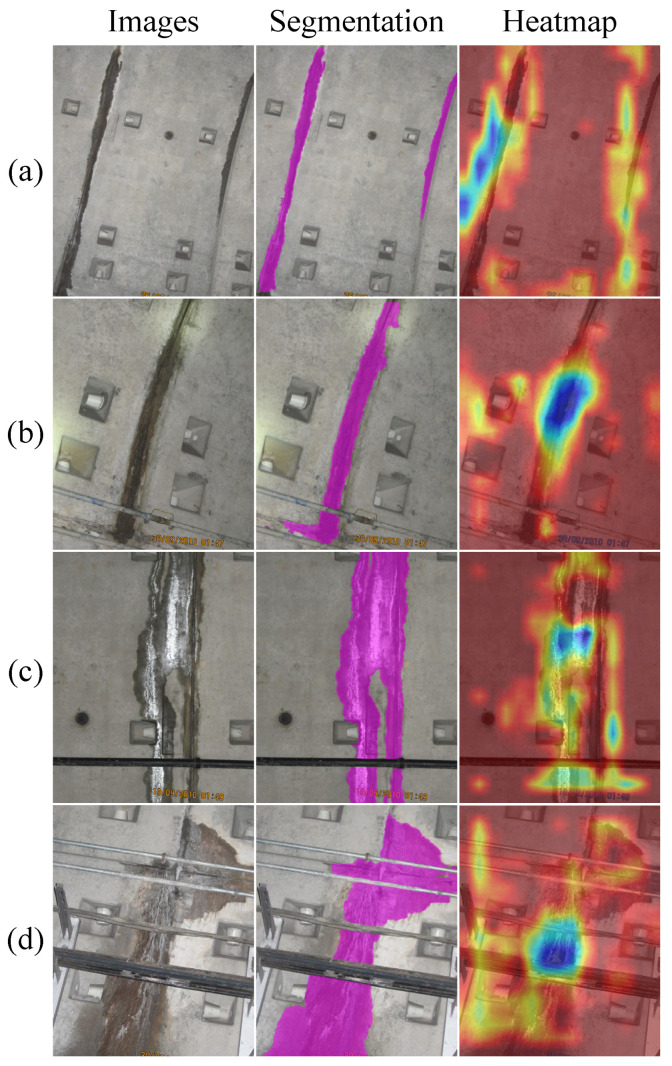
Visualization results of the feature map. Each row (**a**–**d**) represents the processing of different samples. The first column showcases the original tunnel water image that is fed to the model. The second column displays the segmentation results produced by DAEiS-Net, with red areas indicating the recognized water stain regions. The third column presents the model’s feature responses to the input image, with darker colors (closer to blue) representing higher attention of the model on that region.

To further validate the effectiveness of DAEiS-Net, we conducted feature visualization experiments. These experiments intuitively demonstrate the model’s feature responses to different input images, helping to understand how the model identifies and segments areas with tunnel water stains. As illustrated in Figure 11, each row represents the processing of a specific input image, including the original image, segmentation result, and feature heatmap. The first column showcases the original tunnel water image used as input to the model. The second column displays the segmentation results produced by DAEiS-Net, with red areas indicating the recognized water stain regions. The third column presents the model’s feature responses to the input image, where darker colors (closer to blue) represent higher attention from the model to that region. Through analysis, we observed that DAEiS-Net accurately focuses its attention on water stain regions when the stain area is small, as depicted in Figure 11a,b. Conversely, when the stain area is large, as shown in Figure 11c,d, the model’s attention is more dispersed, indicating its strong global perception capability. The feature heatmap illustrates that the model can focus on the overall context when handling large stain areas, ensuring the complete identification of water stain regions. These findings suggest that DAEiS-Net not only possesses strong local feature extraction capabilities but also exhibits excellent global perception abilities. This allows it to flexibly adapt to different scales of water stain scenarios, thereby enhancing the overall performance of tunnel water stain segmentation tasks. In summary, through feature visualization experiments, DAEiS-Net demonstrates outstanding performance in tunnel water stain segmentation tasks, especially in focusing on key areas and handling large stain areas.

In summary, the method proposed in this paper significantly outperforms other relevant methods in addressing issues such as multiscale boundary information loss and global information extraction. Through innovative design of the model structure and optimization of feature extraction mechanisms, the proposed model demonstrates excellent performance in tunnel water stain segmentation tasks. It offers a more robust and efficient solution, highlighting its superiority in addressing challenges associated with tunnel water stain segmentation.

## 5. Conclusions

This paper proposes a novel tunnel water stain segmentation network architecture called Deep Aggregation Network with Edge Information Supplement (DAEiS-Net). Leveraging the classic encoder–decoder structure, the proposed network incorporates DAM and EISM, significantly enhancing the model’s feature representation capability and edge information extraction capacity. Additionally, the decoder part utilizes the SPD module to merge features from different scales, further enhancing the representation capability of edge features. Experimental results demonstrate that DAEiS-Net achieves satisfactory performance on a custom dataset of tunnel water stains. By effectively aggregating multiscale contextual information and extracting edge details, DAEiS-Net successfully tackles the key challenges in tunnel water stain segmentation, demonstrating its significant potential for practical applications.

Future work will focus on further optimizing this model and exploring its application and extension in other related fields. This includes refining the network architecture, improving computational efficiency, and adapting the model to different types of tunnel diseases and other segmentation tasks. Additionally, expanding the dataset to include more diverse tunnel environments and conditions will help to validate and enhance the robustness of DAEiS-Net.

## Figures and Tables

**Table 1:** The specific convolutional settings of the network structure.

Module	Specific Convolutional Settings
Encoding Block 1	[7 × 7, 64, s=2]; [3 × 3, MaxPooling, s=2]
Encoding Block 2	[3 × 3, 64] × 3
Encoding Block 3	[3 × 3, 128] × 4
Encoding Block 4	[3 × 3, 256] × 6
Encoding Block 5	[3 × 3, 512] × 3
DAM	[1 × 1, 256]; [1 × 1, 128] × 2; [1 × 1, 64] × 3
MCAM	[1 × 1, AvgPooling]; [1 × 1, MaxPooling]; [1 × 1, 64] ×2
EISM	[1 × 1, 64]; [1 × 1, 128]; [3 × 3, 64]; [1 × 1, 64]

**Table 2:** Introduction to the TWS dataset.

Item	Description
Dataset Name	TWS (Tunnel Water Stain)
Dataset Purpose	Tunnel water stain segmentation
	Training set: 2000 images
Dataset Size	Validation set: 400 images
	Test set: 100 images
Image Resolution	2330 × 1747; 1944 × 2592
Scenarios and Conditions	Captures various scenarios and conditions of tunnel water leakage
Annotations	Each image has been meticulously annotated, detailing the contours and locations of water leakage areas
	Provides valuable training data for the model,
Annotation Purpose	helping it learn about various shapes,
	and sizes of water stain features

**Table 3:** Experimental results of different models.

Model	Para (M)	mAcc	mIoU	mF1
UNet	33.2	0.5709	0.5608	0.54
PANet	34.9	0.6482	0.6649	0.62
CCNet	40.2	0.7606	0.7556	0.68
Deeplabv3	25.8	0.7772	0.7739	**0.77**
SCDeepLab	42.3	0.7822	0.7274	0.73
ViT-Seg	113.71	**0.8021**	0.7337	0.72
TCDNet	140.1	0.7083	0.6817	0.65
MTISs	19.0	0.7228	0.7005	0.71
**DAEiS-Net (Ours)**	**15.3**	0.7923	**0.7874**	**0.77**

**Table 4:** Results of the ablation experiments.

Baseline	DAM	EISM	SPD	Para (M)	mAcc	Gain (%)	mIoU	Gain (%)	mF1	Gain (%)
✓	-	-	-	13.2	0.7238	/	0.7001	/	0.71	/
✓	✓	-	-	14.8	0.7652	+5.72	0.7537	+7.65	0.71	0
✓	✓	✓	-	15.3	0.7792	+7.65	0.7418	+5.96	0.73	+2.82
✓	-	✓	✓	15.3	0.7856	+8.54	0.7622	+8.87	0.75	+5.63
✓	✓	-	✓	15.3	0.7731	+6.81	0.7521	+7.43	0.74	+4.23
✓	✓	✓	✓	15.3	**0.7923**	**+9.46**	**0.7874**	**+12.46**	**0.77**	**+8.45**

## Data Availability

The data presented in this study are available on request from the corresponding author.
